# Relationships between outdoor physical activity, health-related quality of life, and sleep in 8-to-12-year-old children: an exploratory study

**DOI:** 10.3389/fspor.2025.1516699

**Published:** 2025-03-14

**Authors:** Callista Zayatz, Olivia Hopko, Karlie Gambino, Rocco Paluch, Stephanie Anzman-Frasca, Mackenzie J. Ferrante

**Affiliations:** ^1^ Department of Pediatrics, Jacobs School of Medicine and Biomedical Sciences, University at Buffalo, Buffalo, NY, United States; ^2^ Center for Ingestive Behavior Research, University at Buffalo, Buffalo, NY, United States; ^3^ Department of Nutritional Sciences, School of Environmental and Biological Sciences, Rutgers, The State University of New Jersey, New Brunswick, NJ, United States

**Keywords:** physical activity, outdoor activity, children, sleep, hiking, health-related quality of life

## Abstract

Children today are at high risk of chronic disease partially because of a sedentary lifestyle. High levels of physical activity in children have been linked to increased physical and psychological wellbeing and high sleep quality. Further, time spent outdoors has also been linked to overall wellbeing in children. Outdoor physical activities may have additional benefits for children, especially in winter when indoor sedentary time increases. The present online survey study examined relationships between parent reports of 8-to-12-year-old children's (*n* = 47) general physical activity, hiking frequency, health-related quality of life (a measure of children's physical, psychological, and social wellbeing; HRQoL), and sleep routines in winter 2023. Parent-reported frequency of children's hiking was predictive of their overall wellbeing, where those who hiked more frequently had higher HRQoL scores (*ß* = 1.20, *p* = 0.01, *R*^2^ = 0.24). More frequent hiking was also related to more consistent bedtime and waking routines (*ß* = 0.19, *p* = 0.02, *R*^2^ = 0.27; *ß* = 0.19, *p* = 0.009, *R*^2^ = 0.22). In contrast, overall weekly physical activity level was not predictive of HRQoL scores or sleep*.* The results support further investigation into hiking as a health promotion intervention for youth.

## 1 Introduction

Children between the ages of 8 and 12 years (i.e., preadolescence) are in a unique period of their lives as they transition to having greater independence from their parents/caregivers (1). Multiple factors have placed preadolescents at increased risk for chronic disease, including increasingly sedentary lifestyles and lack of physical activity among this age group (2). In fact, only 25% of children in the United States meet physical activity guidelines (2). Even children who participate in structured sports three times per week fail to meet the recommended 60 min of daily physical activity for adolescents (3). The poor health outcomes related to increased sedentary lifestyles observed in teenagers who do not participate in physical activity (3, 4) suggest the need to identify new areas that encourage physical activity among preadolescents, potentially leading to the development of healthy habits and reduced risk for chronic disease.

Physical activity is an important factor in reducing children's risk for chronic disease and has been linked to cognitive and socioemotional health benefits for children (3). Preadolescents who are physically active may have higher psychological wellbeing (i.e., satisfaction with life) and stronger social connections (i.e., positive relationships with parents and peers) compared with those who are not physically active (4, 5). Previous studies have indicated that children who are more physically active have more consistent bedtime routines and report better sleep quality (6, 7), both of which are important factors for children's health and development. Insufficient and irregular sleep may impact physical wellbeing (7), psychological wellbeing, and academic outcomes for children at a time when they are at increased risk of daytime sleepiness due to extrinsic factors (e.g., biological factors such as puberty); (8) Taken together, increased physical activity among preadolescents may improve many health-related outcomes, including physical wellbeing, psychological wellbeing, and social wellbeing and sleep, for this group.

One type of physical activity that may be especially beneficial for a variety of health behaviors is outdoor physical activity such as hiking (9). Hiking, a form of “green exercise,” combines spending time outdoors with intense physical activity (10) and has been previously linked to reduced stress during the winter among adults (11). Hiking may be particularly beneficial for preadolescents at high risk for sedentary activity, especially during the winter when time spent indoors increases, and is one form of exercise that may have lower barriers to entry compared with traditional physical activities for a number of children (9). Time spent outdoors has been linked to increased physical activity, physical health, psychological health, and sleep among children (12, 13). One study found positive links between families' time spent doing outdoor activities and children's health and wellbeing (14). Another study showed a significant improvement in 9-to-15-year-old children's health-related quality of life (HRQoL; a multidimensional measure of children's physical, psychological, and social wellbeing) among those who participated in nature-based field trips (15). Research on adults suggests that hiking may have greater effects on calmness, anxiety, mood, and stress among adults when compared with walking indoors while viewing scenes of nature (16). Further, green space exposure has previously been associated with an improvement in both sleep quality and quantity (17).

The extant research supports the potential positive impact that nature-based physical activity, including walking outdoors, gardening, and other outdoor physical activity, has on sleep and wellbeing. Although mechanisms for these relationships are still being studied, it is posited that the positive relationship between green space activity and health among adults may be due to sensory-perceptual and immunological processes (18). Two randomized controlled trials conducted by our team in the winters of 2021 and 2022 provided evidence that a hiking intervention that encouraged participants to engage in a winter hiking challenge could improve sleep among adult participants (11, 13). Regardless of the level of physical activity, adults who participate in outdoor physical activity have been observed to have decreased stress, better mental health, and improved sleep, and outdoor exercise may provide a mechanism for parents to connect with children (19, 20).

Compared with research on adults, there is minimal research on the outdoor physical activity of preadolescents, with little being known about the potential benefits that hiking can have on children's health and sleep, especially during times when people generally do not spend time outdoors, such as winter. To begin to fill these gaps and generate hypotheses for future research, the goal of this exploratory study was to examine relationships between physical activities, including hiking, and aspects of 8-to-12-year-old children's health among families who spend time outdoors. The primary objective of this study was to examine relationships between 8-and-12-year-old children's frequency of physical activities in the winter, focusing on frequency of hiking, and aspects of their health-related quality of life. A secondary objective was to examine relationships between these activities and aspects of sleep. We hypothesize that greater frequency of physical activity in the winter, especially hiking, will be associated with higher parent-reported HRQoL for children, higher sleep hygiene, and more consistent sleep-related routines. Complementary to these aims, we explored open-ended responses to two questions about parents' perceptions of the benefits of activity and hiking for their 8-to-12-year-old child.

## 2 Methods

### 2.1 Procedures

A 66-item online survey was assembled by researchers at [University at Buffalo] and comprised of previously developed and validated measures. Adult caregivers (parents and legal guardians; referred to as participants throughout this paper) of 8-to-12-year-old children were recruited for the one-time, online survey study in February and March of 2023. To recruit participants, advertisements were sent as part of the weekly newsletter of and posted to the social media page of a regional hiking group in Western New York. Advertisements specified that participants would be asked to complete an online survey, and upon completion, would be entered into a random drawing to win 1 of 5 $100 gift cards. To be eligible, participants needed to be 18 years or older, be the parent or legal guardian of at least one 8-to-12-year-old child with no health problems precluding participation in physical activity, be English speaking, have online access, live in the Western New York region of the United States, and have, themselves, previously participated in at least one winter hiking challenge. These regional hiking challenges have been offered annually since 2019. After removing ineligible responses that proved to be bots, the screening survey returned 141 responses, of which 62 were deemed eligible to participate. Reasons for exclusion included the following: failure to complete screening questions (*n* = 55), child not in the age range (*n* = 5), not a parent or legal guardian (*n* = 3), and had not completed an eligible winter hiking challenge (*n* = 16). Eligible parties were contacted via email to schedule a short phone call to confirm eligibility. Those who were eligible and completed the phone call (*n* = 49) were sent an individualized link to the online survey, with a request to complete the survey within 1 week, and all surveys were collected by 8 March 2023. Two participants who were sent the link did not complete the survey. Study procedures were approved by the [University at Buffalo] Institutional Review Board.

### 2.2 Measures

The 66-item survey was comprised of previously developed and validated measures to examine participation in outdoor physical activities, HRQoL, and sleep. Some measures were adapted to fit with the current study as described herein.

#### 2.2.1 Demographics

Participants reported their gender, marital status, highest level of education, household income, and number of individuals in the household (both adults and children). Participants also reported on the characteristics of their 8-to-12-year-old child, including gender, race/ethnicity, and whether the child lives in the parent's household. In addition, participants indicated who in the household was the most familiar with the child's daily activities (responses included the following: I am, another parent/guardian, another parent/guardian and I are equally familiar).

#### 2.2.2 Physical activity

Frequency of participation in a variety of physical activities over the previous 7 days was assessed using questions adapted from the Physical Activity Questionnaire for Older Children (PAQ-C) (21). Specifically, participants indicated how often (not at all, 1–2 times, 3–4 times, 5–6 times, and 7 times or more) their child engaged in each of the following activities: hiking/nature walks, other walking exercise, biking, jogging or running, football, soccer, basketball, ice skating, skiing, cross country skiing, and ice hockey. Responses from the PAQ-C were used to create a 7-day activity score. Frequency of participation over the previous 7 days for each of the 12 activities was summed, resulting in a possible composite score ranging from 0 to 60, with 60 indicating a high frequency of participation in many different activities. Participants also reported hours spent being physically active alongside their 8-to-12-year-old child or helping their child be physically active in a typical week using questions adapted from Project F-Eat (22). Response choices included the following: less than 30 min, 30 min–2 h, 2.5–4 h, 4.–6 h, and 6+ h. Frequency of hiking was assessed by asking participants to indicate how often they hiked, how often their child hiked with them, and how often their child hiked in general over the last month (responses included: has not happened in the past 30 days, happened once in the past 30 days, happened a few times in the past 30 days, happened often in the past 30 days, or happened very often in the past 30 days).

#### 2.2.3 Health-related quality of life

The child's physical wellbeing, psychological wellbeing, and autonomy and parent relations were assessed using questions adapted from the KIDSCREEN-27 (5). All questions were asked about the previous 7 days. Physical wellbeing was assessed with the following questions: participants indicated how their child would rate their health (responses included: excellent, very good, good, fair, poor), their ability to be physically active (responses included: not at all, slightly, moderately, very, extremely), and how often their child felt full of energy (responses included: never, almost never, sometimes, almost always, always). To assess psychological wellbeing, parents were asked to report their child's general mood and feelings, including if their child felt life was enjoyable, had been in a good mood, had fun, felt sad, felt lonely, and had been happy with themselves with responses ranging from never, almost never, sometimes, almost always, and always. Finally, to assess autonomy and parent relations, participants were asked to report whether in the last week the child had enough time for themselves as well as if they had been able to do the things they wanted to in their free time (responses included: never, almost never, sometimes, almost always, always). Participants were also asked about their child's feelings of having enough time with their parents, having been treated fairly by their parents, and being able to talk to their parents as needed in the past week (responses included: never, almost never, sometimes, almost always, always). Items from each of the KIDSCREEN-27 subscales physical wellbeing, psychological wellbeing, and autonomy and parent relationships were scored as appropriate and then summed to create composite scores for each subscale (possible ranges include: physical wellbeing, 5–25, with higher scores indicating higher levels of physical wellbeing; psychological wellbeing, 7–35, with higher scores indicating higher levels of psychological wellbeing; autonomy and parent support, 5–25, with higher scores indicating higher levels of parent responsiveness). Final scores from each subscale were summed resulting in a composite HRQoL score (possible range 17–85).

#### 2.2.4 Children's sleep routines and hygiene

Sleep routines were assessed using items and scales from reliable and valid surveys: the Children's Sleep Hygiene Scale (23) and the Children's Sleep Wake Scale (24). Two items from the Children's Sleep Hygiene Scale asked participants to recall whether in the past month their child goes to bed and gets out of bed at around the same time each day, with responses including never, once in a while, sometimes, quite often, frequently, or always. The two items from the Children's Sleep Hygiene Scale were summed to create a sleep hygiene score between 2 and 12, where higher scores indicated consistency in going to bed and rising from sleep. Two scales from the Children's Sleep Wake Scale assessed consistency of bedtime (e.g., going right to bed, making repeated requests, wanting to stay up and do other things, being ready to go to bed at bedtime, and “putting off” going to bed) and consistency of waking (e.g., getting up without help, being ready for the day, being rested and alert, being slow to start in the morning, and being difficult to get out of bed). Responses included: never, once in a while, sometimes, quite often, frequently, or always. Responses from the Children's Sleep Wake Scale subscales, Going to Bed and Returning to Wakefulness, were reverse-scored as appropriate, and mean scores were calculated, resulting in each scale having a range between 1 and 6, where higher scores indicated fewer issues going to bed or rising from sleep.

#### 2.2.5 Benefits of activity (open-ended questions)

To inform future research, participants were also asked to respond to two open-ended questions asking them to describe the following: (1) any benefits they observed from their child participating in outdoor activities and (2) any benefits they see from their child hiking.

### 2.3 Data analysis

Frequencies and means were calculated for participant demographic information and main variables of interest (Table 1). Frequencies were calculated to describe frequency over the previous month of parent hiking, child hiking, and parent and child hiking together. Pearson correlations were conducted to assess relationships between demographic variables and variables of interest.

**Table 1:** Demographic characteristics and responses to variables of interest of participants and their 8-to-12-year-old child (*n* = 47).

Characteristics/responses	Parent/guardian	8-to-12-year-old child
Age (years)^a^, mean (SD)		10.3 (1.3)
Sex^a^, *n* (%)		
Female	37 (82)	17 (38)
Race/ethnicity^a^, *n* (%)		
White		44 (98)
Hispanic		2 (4)
Education^a^, *n* (%)		
Some college/associates	6 (13)	
Bachelor's degree	7 (16)	
Graduate degree	32 (71)	
Marital status^a^, *n* (%)		
Married	38 (84)	
Widowed/divorced	4 (9)	
Annual household income^a^, *n* (%)		
<$49,999	2 (4)	
$50,000–$99,999	14 (31)	
>$100,000	27 (60)	
Parent and child frequency of activity and hiking		
Parent or guardian most familiar with the child's daily activities, *n* (%)		
Myself	31 (69)	
Another parent/guard	1 (2)	
Both equally familiar	13 (29)	
Amount of free time spent being active in previous 7 days, *n* (%)		
Very often/quite often	4 (9)	
Often/sometimes	26 (55)	
Very little time	17 (36)	
No. of different activities in the previous 7 days, mean (SD)		2.8 (1.2)
Hiking, *n* (%)		21 (45)
Frequency of hiking in previous 30 days^a^, *n* (%)		
Never	8 (17)	12 (26)
A few times (1–3)	23 (50)	24 (52)
Often (1/week)	10 (22)	4 (15)
Very often (>1/week)	5 (11)	6 (13)
Frequency of hiking together (parent and child) in previous 30 days^a^, *n* (%)		
Never		14 (54)
A few times (1–3)		24 (52)
Often (1/week)		4 (15)
Very often (>1/week)		4 (15)

^a^ *n* = 45 or *n* = 46 due to participants not completing the entire survey.

To address the objectives of the study, linear regression models tested: (1) if frequency of participation in outdoor physical activities in general and frequency of hiking specifically predicted overall HRQoL and HRQoL subscales; and (2) if frequency of participation in outdoor physical activities in general and frequency of hiking specifically predicted children's sleep routines and sleep hygiene. Children's age, gender, and race, and parent's education level were considered covariates in the models. Data were analyzed using SAS v 9.4 (IBM Corp).

Open-ended responses, in which parents described the benefits they observed from their child participating in outdoor physical activities, were independently coded by CZ and KG to summarize recurring themes. Once complete, the themes and coded data were compared, and in instances where responses did not agree, researchers discussed until consensus was reached. The percent of participants endorsing each theme was calculated by CZ and confirmed by KG. All participant names were changed to protect the privacy of the participants.

## 3 Results

Frequencies and means for participant demographic information and main variables of interest are presented in Table 1. A majority of participants (*n* = 47) were highly educated (71% obtained a graduate degree), white (98%), and female (82%). Many parents reported that their child “sometimes, often, or very often” did outdoor physical activities in their free time (66%), with almost half of parents reporting that hiking was one of those activities in the previous 7 days (*n* = 21, 46%). Most parents reported hiking at least once with their child in the previous 30 days (*n* = 32, 70%), with 17% (*n* = 8) reporting hiking at least weekly with their child over the previous 30 days.

There were significant, positive correlations between parent report of children's hiking from the PAQ-C and the other child frequency of hiking variables (child hiking frequency: *r* = 0.52, *p* < 0.001; parent and child hiking together frequency: *r* = 0.52, *p* < 0.001). There were also significant, positive correlations between children's overall PAQ-C score and children's frequency of hiking variables (child hiking frequency: *r* = 0.51, *p* < 0.001; parent and child hiking together frequency: *r* = 0.35, *p* = 0.02).

There were significant negative associations between parent education level and the hiking variables, where higher education was moderately associated with a lower frequency of children's hiking (*r* = −0.32, *p* = 0.03) or parents and children hiking together (*r* = −0.43, *p* < 0.005). Education was also moderately negatively associated with children's sum scores on the KIDSCREEN-27 Psychological Wellbeing Scale (*r* = −0.31, *p* = 0.04).

### 3.1 Health-related quality of life, hiking, and activity

On average, HRQoL scores were relatively high in this sample (overall HRQoL, x = 66.8, SD = 7.5; physical wellbeing, x = 19.9, SD = 3.4; psychological wellbeing, x = 28, SD = 3.3; parent relationships and autonomy, x = 18.9, SD = 2.5). More frequent hiking by children was predictive of higher overall HRQoL scores (*ß* = 1.20, *p* = 0.01, *R*^2^ = 0.24) and physical wellbeing (*ß* = 0.72, *p* < 0.001, *R*^2^ = 0.34), but not psychological wellbeing (*p* = 0.14, *R*^2^ = 0.22) or parent relationships and autonomy (*p* = 0.30, *R*^2^ = 0.07). Higher parent-reported frequency of parents and children hiking together was predictive of children's overall HRQoL scores (*ß* = 1.56, *p* = 0.005, *R*^2^ = 0.27), physical wellbeing (*ß* = 0.79, *p* = 0.002, *R*^2^ = 0.31), but not parent autonomy and relationships (*p* = 0.09, *R*^2^ = 0.12) or psychological wellbeing (*p* = 0.08, *R*^2^ = 0.24). Children's 7-day activity score was not a statistically significant predictor children's overall HRQoL scores (*p* = 0.40. *R*^2^ = 0.12), physical wellbeing (*p* = 0.08, *R*^2^ = 0.18), parent relationships and autonomy (*p* = 0.99, *R*^2^ = 0.05), or psychological wellbeing (*p* = 0.88, *R*^2^ = 0.18).

### 3.2 Sleep, hiking, and activity

On average, parent report of children's sleep consistency indicated few issues for both going to bed (x = 4.1, SD = 1.3) and rising from sleep (x = 4.3, SD = 1.1). Parents also reported sleep hygiene scores close to the midpoint (x = 7.4, SD = 1.0). More frequent hiking by children was predictive of more consistency in sleep as measured by the going to bed scale (*ß* = 0.19, *p* = 0.02, *R*^2^ = 0.27) and rising from sleep scales (*ß* = 0.19, *p* = 0.009, *R*^2^ = 0.22) but was not predictive of children's sleep hygiene scores (*p* = 0.32, *R*^2^ = 0.09). Similar relationships were seen across frequency of parents and children hiking together (going to bed: *ß* = 0.24, *p* = 0.01, *R*^2^ = 0.28; rising from sleep *ß* = 0.26, *p* = 0.002, *R*^2^ = 0.28; Hygiene *p* = 0.42, *R*^2^ = 0.08). Children's 7-day score was not predictive of sleep consistency (going to bed: *p* = 0.99, *R*^2^ = 0.15; rising from sleep: *p* = 0.82, *R*^2^ = 0.07) or sleep hygiene (*p* = 0.54, *R*^2^ = 0.07).

### 3.3 Additional benefits of hiking for children

Emergent themes of open-ended question responses included parents’ belief that as a result of participating in outdoor physical activities, their 8-to-12-year-old child was healthier and got along better with others (23.4%). For example, one participant reported “Just a better well-being. Hiking has given him the opportunity to visit a great deal of amazing places.” Another participant endorsed increased cooperation along with improved mood of their child, stating “When [childname] gets time outdoors, her mood improves, she is more cooperative and helpful.” Many participants also endorsed an improvement in their child's mood, with answers ranging from their child being happier to calmer to both (42.6%): “His mood improves and he seems to be calmer”; “She seems happier and she communicates better.” Fewer parents noted that their child was more focused or that they had improved sleep as a result of outdoor physical activities (12.7%). One participant who noted multiple benefits: “Observing and appreciating details nature. Provides time together as a family. Sleeping better at night. Sparks creativity.” Another less reported theme was that their child gained confidence (19.1%): “[childname] has gained a ton of confidence from hiking specifically. Learning to navigate in the woods and to do something really hard has been helpful.”

### 3.4 Additional benefits of hiking for families

Similar themes emerged emphasizing familial involvement in hiking and barriers to doing so. One noted that their child spending time outdoors is “a chance to have conversations [with me] and he likes to sit and be peaceful in nature.” Another participant reported that their child “tends to gravitate towards being outside, in her free time, as a result. When I'm present, it's a great opportunity for conversation.” A few participants endorsed liking the structure of hiking and motivation to continue doing so (12.7%), while others described their children's increased desire to hike independently (12.7%). A few barriers were noted, but they included difficulties finding time to hike due to factors such as weather, injuries, or logistics (14.9%). An example is “My son loves hiking but when our younger kids are in strollers it makes locations hard to go without carrying them and my body doesn't allow that anymore.” Notably, one person reported “When COVID began, I made my children hike every single day. Now that we are back into our normal routines again it is harder to find the time […] He whines a bit when it is cold out, but most of the time he enjoys it.”

## 4 Discussion

Children in the United States are at high risk for chronic disease due to recent increases in sedentary lifestyles and screen time and not meeting physical activity recommendations (4). Among both adults and teenagers, outdoor physical activity has been linked with a reduced risk of chronic disease, overall high quality of life (e.g., physical and psychological wellbeing), and better sleep quality and quantity (12, 14, 16, 17, 25). The results of the present, exploratory study support that these links may also be true for preadolescents, where hiking specifically may support children's HRQoL, and highlight the need for future research in this area.

Among adults, benefits of outdoor physical activity have been observed regardless of the level of physical activity. Consistent with our hypothesis, hiking was associated with more consistency in sleep, physical, and overall wellbeing. However, overall frequency of physical activity was not associated with these outcomes. It is important to note that the population represented by our participants were already highly active and started with higher HRQoL scores. All parents indicated that their child participated in at least one physical activity in the previous 7 days, with the average number of individual activities for the present population being three different activities over the previous 7 days. This is higher than is typically seen among other studies examining physical activity in children. A 2012 study found that one-third of teenage girls reported their down time as involving screen-based behaviors (7). A 2015 study found that only 21% of boys and 16% of girls met physical activity recommendations for their age range (7, 26). Findings from the present study, in combination with previous research, suggest that the facilitation of a nature-based physical activity, such as hiking, may be beneficial for health, regardless of one's baseline level of activity.

Previous studies have noted that parental health and stress can impact relationships with preadolescents (20). This is important, as it suggests the need for interventions that can foster positive parent–child interactions during preadolescence (27). Levels of parent (in)activity are also directly correlated with children's activity levels (28). While the present study did not find a significant relationship between parents and children hiking together and parent relationships and autonomy, several parents in the present study pointed out that hiking offered an opportunity for one-on-one conversations that they may not otherwise have had with their child. Almost half of parents reported that their children were getting along better with others and showed improved moods. Data from the present study show that on average, children in this study started out at higher parent relationships and autonomy score (x = 18.8/25; data not given). This may be because this survey was completed by parents and children may feel differently about their relationships than their parents perceive. Outdoor hiking interventions may provide an easy, low-cost solution to strengthening parent–child relationships, although future research could study this more explicitly. The results from the present study also indicate links between higher parent educational attainment and lower frequency of children's hiking and scores for children's psychological wellbeing. Parents with higher educational attainment and higher income may have greater access to indoor activities in the winter, which may take the place of winter hiking. Parents in the present study indicated that their children were participating in a number of indoor activities across the previous 30 days, many of which tend to be more costly (e.g., swimming, taekwondo, etc.; data not given), which may also play a role in decreased hiking for some members of the study population.

In addition to supporting the hypothesis that hiking frequency may be linked to children's physical and psychological health, the results from the present study linked frequent hiking to more consistent sleep routines. A relationship between hiking and better sleep also emerged as a theme within the open-ended questions where many parents noted that activity and hiking led to better sleep for their 8-to-12-year-old children. Sleep is a risk factor for chronic disease in preadolescents, and prior work has negatively linked increased screen time with sleep (26, 29). Prior work has indicated that children who are more physically active have more consistent bedtime routines and report better sleep quality in general (6, 7). Furthermore, there are links between the amount of green space in one's neighborhood and sleep duration, where more green space has been equated with longer sleep duration (30). Taken together, coupling physical activity with nature may have potential for the greatest benefit on one's sleep. Currently, there is little research linking either physical activity or outdoor physical activity and sleep, especially among adolescents. The results from the present exploratory study demonstrate that there may be potential for future studies to explore these links further and test the effects of hiking and other outdoor activities on sleep among adolescents.

The limitations of this study include the fact that the parent reported on behalf of their child, which may introduce bias. The present survey was also completed retrospectively, asking about the previous 7- and 30-day periods, which may have introduced recall bias. Shared methods variance is also a concern, with all measures being completed by the same respondent. While concerns about bias may be tempered by prior psychometric research demonstrating validity of the present measures (5, 21–24, 31, 32), future research could expand on the present study by examining links between hiking, sleep, and HRQoL using gold standard measurements for physical activity and sleep (e.g., actigraphy data) among adolescents using self-reports of HRQoL in place of proxy-reports. Despite open-ended statements suggesting that hiking may reduce familial stress, without parent stress having been directly measured, no definitive conclusions beyond that of what parents reported for their child can be drawn. Participants were recruited online using the social media site of a regional hiking group, possibly introducing selection bias, given that they may be more likely to hike at baseline and view this activity as having a more positive impact on their child, thus limiting the generalizability of our results. Our inclusion criteria required that the reporting parent have engaged in at least one winter hiking challenge but did not necessitate this for 8-to-12-year olds. The study results showed that 17% of parents and 26% of children had not hiked at all in the previous 30 days, suggesting that the level of hiking engagement across the population may be more generalizable. The cold climate in Western New York during winter may be viewed as a limitation, given that it may be more difficult to access hiking due to weather conditions, although previous research suggested that hiking interventions completed in the winter are feasible (17, 19). People living in year-round temperate climates may particularly benefit from hiking interventions, given the weather would permit greater opportunities to hike.

The current study is intended to be hypothesis-generating, building off our own previous research (11, 19) and highlighting important gaps. A 2024 paper from Lesser et al. explored the potential for green space activities, such as hiking, as a way to engage diverse populations in a physical activity with few barriers to entry (9). Hiking is seen as one such activity—it is inexpensive and requires little skill and is accessible to many. Taken together, the results from the present study and previous work highlight future research that could provide interventions to engage new populations in hiking and examine links between outdoor physical activity, HRQoL, and sleep among broader populations.

In conclusion, preadolescence is a crucial time as children have increased independence and there have been vast increases in screen time use and time spent being sedentary in this age group. In the present study, 8-to-12-year-old children's hiking was predictive of their physical and overall wellbeing and sleep consistency. When parents reported hiking together with their children, positive associations with physical wellbeing and sleep were observed. Previous literature has suggested that hiking is a feasible intervention to improve overall health and sleep among adults, and the results of this study demonstrate that these health benefits may be generalizable to preteens as well. Hiking encourages physical activity outdoors, which may be especially useful when people generally spend more time indoors during winter. Hiking may be a way to prevent the development of chronic disease among preadolescents and may also promote positive relationships between youths and their parents, highlighting opportunities for future studies to test these ideas among a broader sample of preadolescents.

## Data Availability

The raw data supporting the conclusions of this article will be made available by the authors without undue reservation.
